# Bleaching Devitalized Teeth with Pyrozone

**Published:** 1900-01

**Authors:** J. P. Parker

**Affiliations:** Santa Cruz, Cal.


ARTICLE II.

### BLEACHING DEVITALIZED TEETH WITH PYROZONE.

J. P. FARKER, D. D. S., SANTA CRUZ, CAL.

This is a subject less written of than practiced, which I think it well to bring before the profession for discussion, that ideas may be brough to light, giving us all the benefit of them.

Why should we bleach a discolored tooth? Nature gives us our teeth with a uniformity of color. With the death of a pulp comes a change, more or less discoloring the affected tooth, and in some instances making it very noticeable to the casual observer and objectionable to the possessor. For instance, when we meet a person with nice, clean, white teeth, we observe nothing wrong, the eye not being attracted to any especial feature; but let there be one decidedly dark tooth in the front of the mouth, and the eyes are so involuntarily fixed as to require an effort on the part of the observer to refrain from gazing offensively. So, to have all the features in harmony, the teeth must be harmonious.

When should we bleach? It has been the practice of many of our best operators to rid themselves and their patients of a badly discolored tooth by removing the natural crown and replacing it with an artificial one, thus making it expensive, and giving the patient a tooth which will never be quite as perfect in strength and harmony as Nature gave, for there are few instances when the artificial becomes an improvement over the natural.

The time consumed and the inconvenience to the patient in bleaching, as a rule, is less than in crowning. In bleaching the tooth and root are made more aseptic, thus improving the sanitary conditions of the mouth, and thereby lessening the chances of pericemental inflammation. In bleaching with pyrozone we remove septic matter that we would not if we were only crowning. A tooth should always be bleached instead of crowned, unless the crown will look better and be stronger.

I begin the process of bleaching by first placing the rubber as closely as possible around three or more teeth; if there is a cavity of decay, excavate that and open the pulp-canal; then with a spray of a 25 per cent. solution of pyrozone reach all exposed points inside and out, following with blasts from the hot-air syringe. When it is difficult to reach far into the root with the spray, I employ cotton to carry pyrozone to the end of the root. One need not fear any serious results in so doing; and yet, if there is no necessity for so doing, it is well to avoid crowding it through the apical foramen. After continuing this treatment from twenty to forty minutes, close up the tooth, and in forty-eight hours it will present a great change, usually becoming as white as the others. However, if it does not, repeat the treatment, and it will be a rare case that will need a third application.

I find it very difficult to determine at the first sitting, and while operating, to what extent the pyrozone has affected the tooth, some cases being much more stubborn than others, and in some it is hard to see any improvement at the time of the operation, but the next day may bring great changes.

There are two kinds of stain that I have found very hard to remove, and I doubt if pyrozone will effect a satisfactory result. The first is a mineral stain, and the second is occasioned by the tooth having been stained with oil of cassia or cinnamon.

One writer speaks of pyrozone as injurious to the vital teeth adjoining the one to be bleached, and suggests covering them with wax, but I never have seen any necessity for such a precaution; he also advises cleansing the teeth with alcohol before beginning to bleach, but I see no necessity for that. Another writer speaks of pyrozone 25 per cent. as a *caustic*, spelled with capitals, and yet, it is the most yielding of all caustics, as it does not destroy tissue. True it burns for a time, but the tissue will live, and in a few hours be as though it had never been burned. This writer also speaks of continuing the operation for three hours in stubborn cases, but I see no necessity for it.

Ladies and gentlemen, the time is past when we can cut away a good crown just because it is discolored; we must have some other excuse, and what was good practice ten years ago may be malpractice to-day; so let us move forward with the times, and improve every opportunity to advance; and now that we have had for four years means at our hands for a simple, easy, harmless and inexpensive method of bleaching, it is high time we should use it. It may interfere somewhat with the sale of crown teeth, but it will be much better for our patients and our pockets in the end.

### DISCUSSION.

Dr. Clyde Payne: Mr. President, my experience in the use of pyrozone in bleaching teeth has been somewhat limited, though I have bleached several. I think that it is considered the best, and is practically the only means that we have for bleaching teeth permanently, and without damage to the tooth structure.

Dr. H. W. Moore: I may say that in bleaching teeth I have been successful sometimes by repeating the application. I have made a success of it by adjusting the rubber-dam over the tooth, tying it over firmly, turning it up, making a pouch of it, and pouring in a 25 per cent. solution of pyrozone. Tie it firmly in this position and bring down the ends, just allowing the liquid to remain in contact with the tooth for an hour or so. I have succeeded in bleaching teeth in this way when I have failed in another.

Dr. A. F. Merriman, Jr.: I think the suggestion of Dr. Moore a good one. I think I accomplish the same thing without putting my patient to rest for an hour. I had an experience recently that pleased me very much. The patient had met with an accident some twenty-five years ago. She received a blow upon the central and lateral incisors which I judge was the indirect cause of the discolored teeth. I found it necessary to crown the central, but the lateral I bleached to my satisfaction. After the removal of the debris, I used ammonia, and instead of the 25 I employed the 5 per cent. pyrozone, to my perfect satisfaction, for I found I had a good color. The tooth had been discolored twenty five years. I have recently seen it, and there is no re-discoloration. I think you will find that the 5 per cent. solution used with the hot-air blast is about as efficient as the 25 per cent. and much more easily handled.

Dr. C. E. Post: Mr. Chairman, I would like to ask the gentlemen if they are always successful in permanently bleaching? No doubt most of us are able to restore the color to a tooth at the time of the operation, but does it remain that way? Is that your experience, Dr. Parker?

Dr. Parker: Mr. President, perhaps I can answer that best by citing a case. Three years ago a young man came to me for some slight operation, and I found a very much discolored tooth. I think it was the right central. I remarked that I could restore that tooth to its normal color, and he suggested that I try it, which I did. Last spring I had the pleasure of meeting the same gentleman. He and his wife were treveling through the country. That operation was so satisfactory that he, and his wife also, reported at the office for what dentistry each required at that time. I could mention a number of other cases, but will say that with the exception of one, in which I employed the 5 per cent. solution, I know of but one tooth that has become re-discolored. They have all retained their bleached condition, and it would be difficult to distinguish them from their neighbors.

Dr. Post: Will you tell us what you do after bleaching?

Dr. Parker: Just as I would do after removing the pulp; simply pack the canal with gutta percha, and insert any filling that I think best in the remaining cavity.

Dr. Post: Mr. President, I am glad to hear this very favorable report from Dr. Parker on the use 25 per cent. pyrozone. My experience, however, has been rather varied. Dr. Kirk some time ago recommended the use of peroxide of sodium. He suggested making a 50 per cent. solution of sodium. I got a box of the peroxide of sodium three or four years ago, and tried making the 50 per cent. solution; afterwards I simply placed the powder in the tooth and applied some water to it, making two or three applications of that kind very satisfactorily. My experience has been more satisfactory in the use of peroxide of sodium than in the use of peroxide of hydrogen or pyrozone.

Dr. Goddard: Mr. President, I merely want to add a word of caution; do not carry the bleaching too far. I bleached a lateral incisor for a young lady of about eighteen. When I finished, the tooth was actually whiter than its neighbors, and was so the last time I saw her.

Dr. Platt: Mr. President, I would like to say in relation to what Dr. Goddard has just said, I met with the same experience not long ago. It is about a month since I bleached the tooth and got it too white, but it has now pretty nearly reached its normal color. I think it acts very much as a natural inlay would. You may put in an inlay and have it too light at first, but in a few days it will assume the color of the adjoining teeth. Why it does so, I don't know. I think that principle applies to a tooth that has been bleached very thoroughly, dried out perhaps. I would like to say also that in the use of pyrozone, the 25 per cent. the cataphoric procedure is of very great assistance. I have in teeth that were very much discolored taken out the putrescent pulp and put an amalgam filling in, and in forty-five minutes to an hour and a half—at the longest two hours—I have bleached those teeth white enough for every purpose, so they could not be very readily detected from the adjoining teeth. In one case I bleached a lateral incisor that was very badly discolored in thirty five minutes with the cataphoric apparatus and a 25 per cent. solution of pyrozone. It was as near normal as you could possibly expect a devitalized tooth to become. The use of the cataphoric apparatus with pyrozone greatly expedites the procedure. So far as I can find, it is without any pain at all where the tooth is devitalized. Two or three patients have complained of a tingling sensation where the negative pole was placed, but no pain. It hastens the work very materially, and does not, so far as I can determine, do any injury to the teeth.

Dr. Goddard: I will ask Dr. Parker if he has any more difficulty in bleaching a tooth which is discolored yellow than one which is blue? I have a case at present which is yellow in color, and I have not been able to bleach it,

Dr. Parker: In answering I must say I have not observed any difference. It has never before been called to my attention.

Dr. Whitney: I would ask Dr. Parker how he proceeds when he has a metal stain to remove? I find the greatest difficulty in bleaching that class of cases—metallic stains.

Dr. Parker: I know of nothing that will remove a metallic stain from tooth structure, as, for instance, nitrate of silver or any thing of that sort.

Dr. Post: I think the peroxide of sodium will remove the stains of amalgam.

Dr. O'Connor: Mr. President, Dr. Parker stated in paper that he found oil of cassia produced one of the most difficult stains to bleach. If the doctor will try a solution of dehydrated turpentine, which can be had at the wholesale drug houses, I think he can most effectually remove the stain.—*Pacific Stomatological Gazette.*

